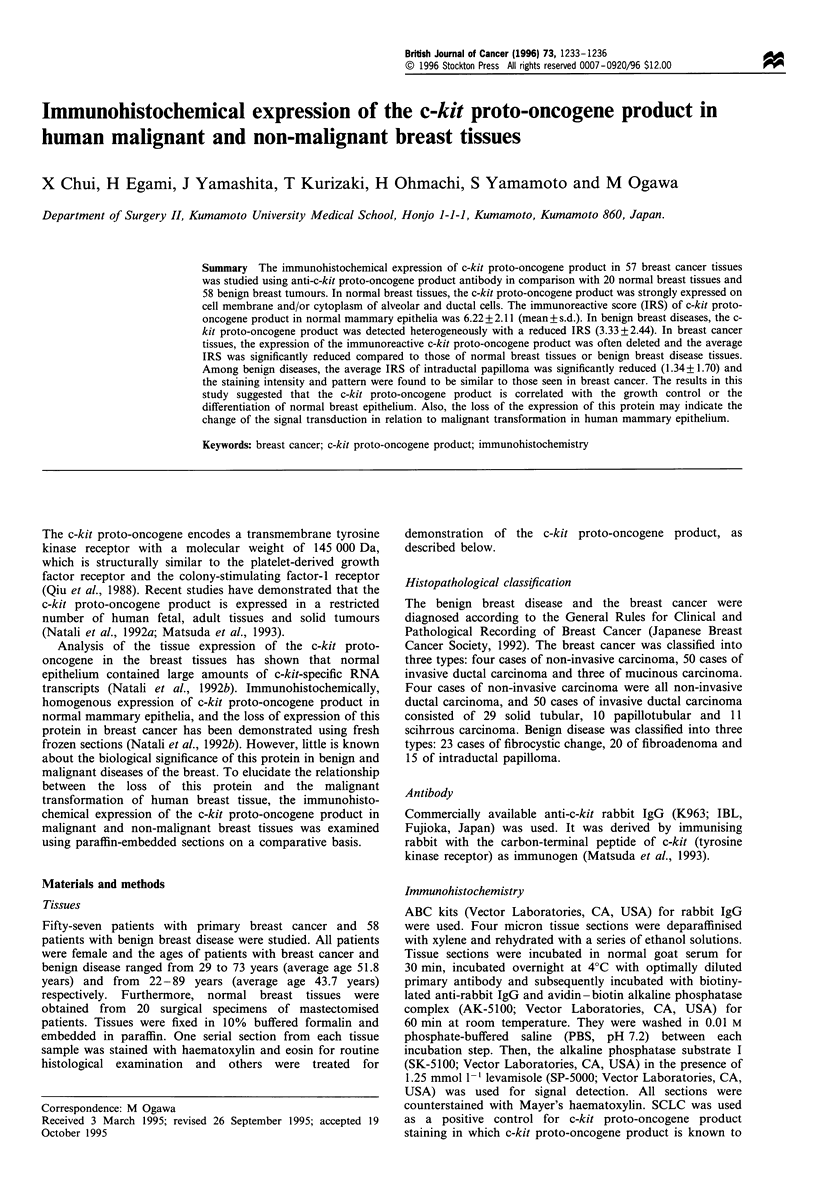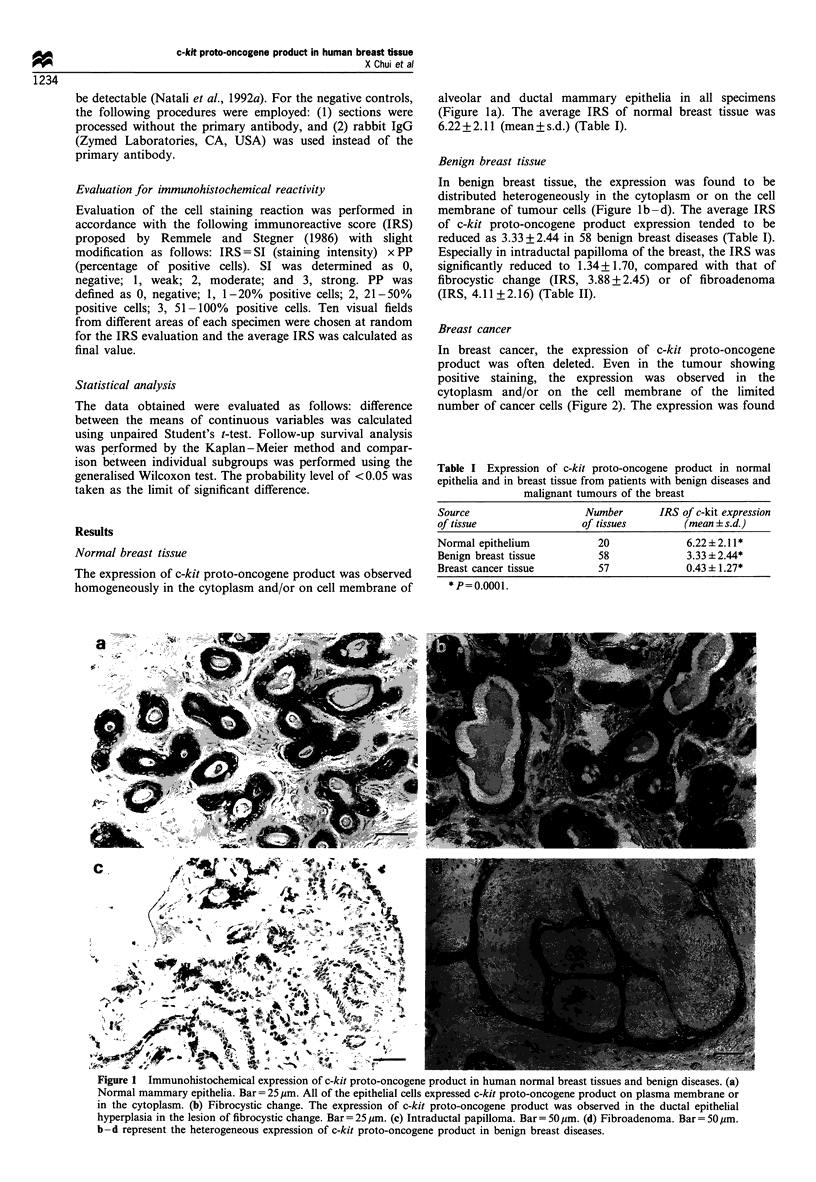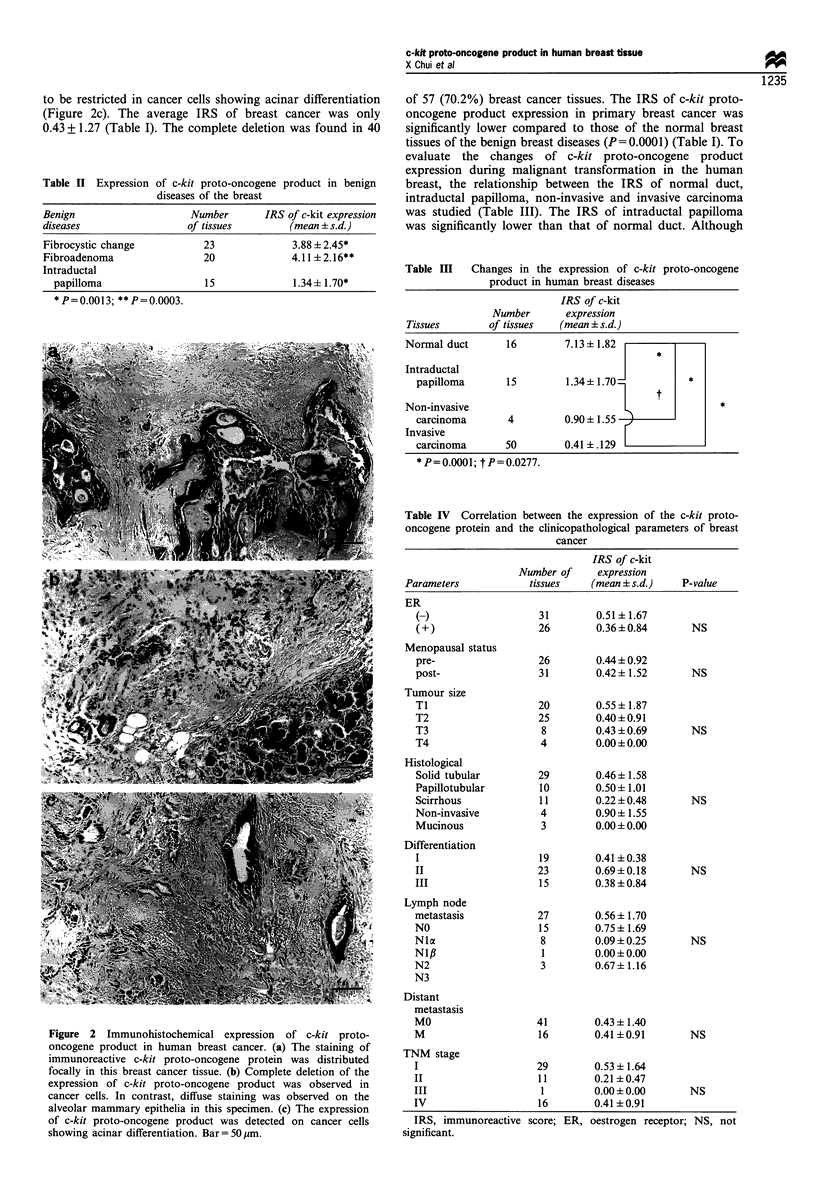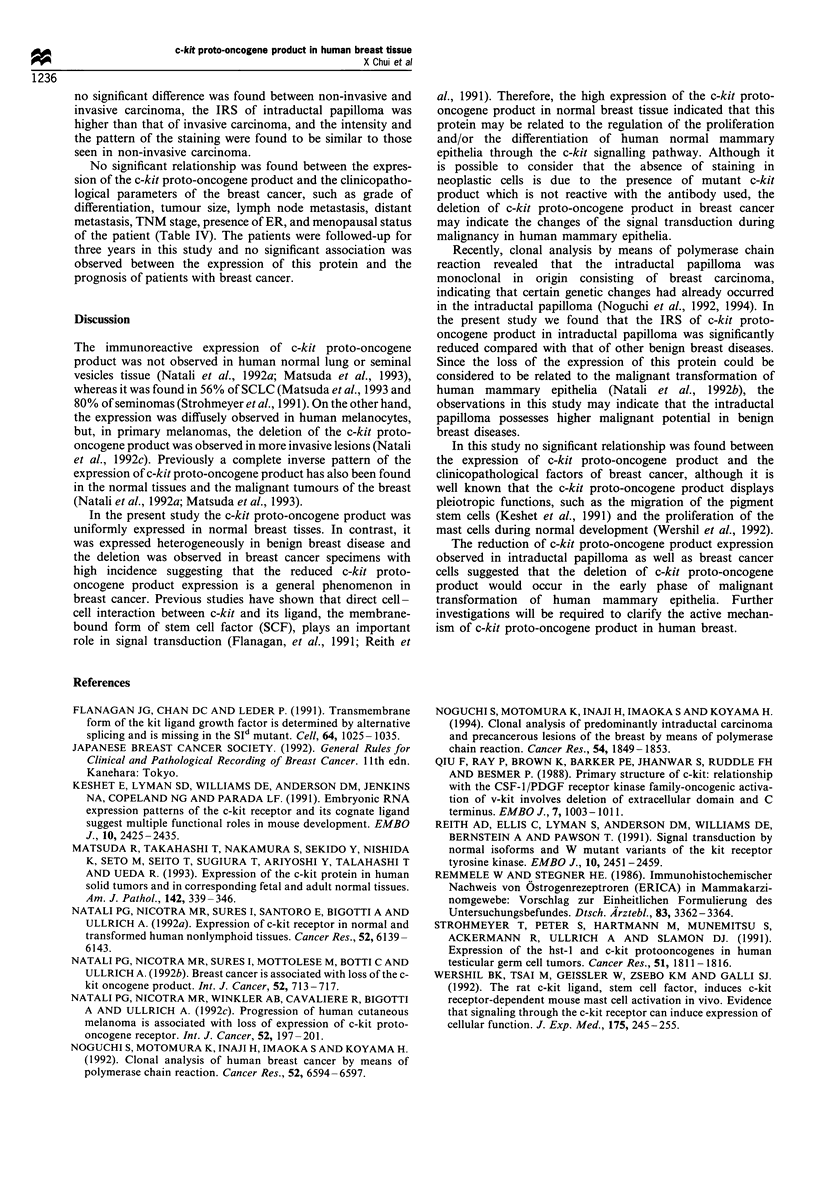# Immunohistochemical expression of the c-kit proto-oncogene product in human malignant and non-malignant breast tissues.

**DOI:** 10.1038/bjc.1996.236

**Published:** 1996-05

**Authors:** X. Chui, H. Egami, J. Yamashita, T. Kurizaki, H. Ohmachi, S. Yamamoto, M. Ogawa

**Affiliations:** Department of Surgery II, Kumamoto University Medical School, Japan.

## Abstract

**Images:**


British Journal of Cancer (1996) 73, 1233-1236
© 1996 Stockton Press All rights reserved 0007-0920/96 $12.00

### Immunohistochemical expression of the c-*kit* proto-oncogene product in human malignant and non-malignant breast tissues

X Chui, H Egami, J Yamashita, T Kurizaki, H Ohmachi, S Yamamoto and M Ogawa

*Department of Surgery II, Kumamoto University Medical School, Honjo 1-1-1, Kumamoto, Kumamoto 860, Japan.*

**Summary** The immunohistochemical expression of c-*kit* proto-oncogene product in 57 breast cancer tissues was studied using anti-c-*kit* proto-oncogene product antibody in comparison with 20 normal breast tissues and 58 benign breast tumours. In normal breast tissues, the c-*kit* proto-oncogene product was strongly expressed on cell membrane and/or cytoplasm of alveolar and ductal cells. The immunoreactive score (IRS) of c-*kit* proto-oncogene product in normal mammary epithelia was $6.22 \pm 2.11$ (mean $\pm$ s.d.). In benign breast diseases, the c-*kit* proto-oncogene product was detected heterogeneously with a reduced IRS ($3.33 \pm 2.44$). In breast cancer tissues, the expression of the immunoreactive c-*kit* proto-oncogene product was often deleted and the average IRS was significantly reduced compared to those of normal breast tissues or benign breast disease tissues. Among benign diseases, the average IRS of intraductal papilloma was significantly reduced ($1.34 \pm 1.70$) and the staining intensity and pattern were found to be similar to those seen in breast cancer. The results in this study suggested that the c-*kit* proto-oncogene product is correlated with the growth control or the differentiation of normal breast epithelium. Also, the loss of the expression of this protein may indicate the change of the signal transduction in relation to malignant transformation in human mammary epithelium.

**Keywords:** breast cancer; c-*kit* proto-oncogene product; immunohistochemistry

The c-*kit* proto-oncogene encodes a transmembrane tyrosine kinase receptor with a molecular weight of 145 000 Da, which is structurally similar to the platelet-derived growth factor receptor and the colony-stimulating factor-1 receptor (Qiu *et al.*, 1988). Recent studies have demonstrated that the c-*kit* proto-oncogene product is expressed in a restricted number of human fetal, adult tissues and solid tumours (Natali *et al.*, 1992*a*; Matsuda *et al.*, 1993).

Analysis of the tissue expression of the c-*kit* proto-oncogene in the breast tissues has shown that normal epithelium contained large amounts of c-*kit*-specific RNA transcripts (Natali *et al.*, 1992*b*). Immunohistochemically, homogenous expression of c-*kit* proto-oncogene product in normal mammary epithelia, and the loss of expression of this protein in breast cancer has been demonstrated using fresh frozen sections (Natali *et al.*, 1992*b*). However, little is known about the biological significance of this protein in benign and malignant diseases of the breast. To elucidate the relationship between the loss of this protein and the malignant transformation of human breast tissue, the immunohistochemical expression of the c-*kit* proto-oncogene product in malignant and non-malignant breast tissues was examined using paraffin-embedded sections on a comparative basis.

### Materials and methods

### *Tissues*

Fifty-seven patients with primary breast cancer and 58 patients with benign breast disease were studied. All patients were female and the ages of patients with breast cancer and benign disease ranged from 29 to 73 years (average age 51.8 years) and from 22-89 years (average age 43.7 years) respectively. Furthermore, normal breast tissues were obtained from 20 surgical specimens of mastectomised patients. Tissues were fixed in 10% buffered formalin and embedded in paraffin. One serial section from each tissue sample was stained with haematoxylin and eosin for routine histological examination and others were treated for demonstration of the c-*kit* proto-oncogene product, as described below.

### *Histopathological classification*

The benign breast disease and the breast cancer were diagnosed according to the General Rules for Clinical and Pathological Recording of Breast Cancer (Japanese Breast Cancer Society, 1992). The breast cancer was classified into three types: four cases of non-invasive carcinoma, 50 cases of invasive ductal carcinoma and three of mucinous carcinoma. Four cases of non-invasive carcinoma were all non-invasive ductal carcinoma, and 50 cases of invasive ductal carcinoma consisted of 29 solid tubular, 10 papillotubular and 11 scihrrous carcinoma. Benign disease was classified into three types: 23 cases of fibrocystic change, 20 of fibroadenoma and 15 of intraductal papilloma.

### *Antibody*

Commercially available anti-c-*kit* rabbit IgG (K963; IBL, Fujioka, Japan) was used. It was derived by immunising rabbit with the carbon-terminal peptide of c-*kit* (tyrosine kinase receptor) as immunogen (Matsuda *et al.*, 1993).

### *Immunohistochemistry*

ABC kits (Vector Laboratories, CA, USA) for rabbit IgG were used. Four micron tissue sections were deparaffinised with xylene and rehydrated with a series of ethanol solutions. Tissue sections were incubated in normal goat serum for 30 min, incubated overnight at 4°C with optimally diluted primary antibody and subsequently incubated with biotinylated anti-rabbit IgG and avidin-biotin alkaline phosphatase complex (AK-5100; Vector Laboratories, CA, USA) for 60 min at room temperature. They were washed in 0.01 M phosphate-buffered saline (PBS, pH 7.2) between each incubation step. Then, the alkaline phosphatase substrate I (SK-5100; Vector Laboratories, CA, USA) in the presence of 1.25 mmol $l^{-1}$ levamisole (SP-5000; Vector Laboratories, CA, USA) was used for signal detection. All sections were counterstained with Mayer's haematoxylin. SCLC was used as a positive control for c-*kit* proto-oncogene product staining in which c-*kit* proto-oncogene product is known to

Correspondence: M Ogawa
Received 3 March 1995; revised 26 September 1995; accepted 19 October 1995

be detectable (Natali *et al*., 1992*a*). For the negative controls, the following procedures were employed: (1) sections were processed without the primary antibody, and (2) rabbit IgG (Zymed Laboratories, CA, USA) was used instead of the primary antibody.

*Evaluation for immunohistochemical reactivity*

Evaluation of the cell staining reaction was performed in accordance with the following immunoreactive score (IRS) proposed by Remmele and Stegner (1986) with slight modification as follows: IRS = SI (staining intensity) × PP (percentage of positive cells). SI was determined as 0, negative; 1, weak; 2, moderate; and 3, strong. PP was defined as 0, negative; 1, 1–20% positive cells; 2, 21–50% positive cells; 3, 51–100% positive cells. Ten visual fields from different areas of each specimen were chosen at random for the IRS evaluation and the average IRS was calculated as final value.

*Statistical analysis*

The data obtained were evaluated as follows: difference between the means of continuous variables was calculated using unpaired Student's *t*-test. Follow-up survival analysis was performed by the Kaplan–Meier method and comparison between individual subgroups was performed using the generalised Wilcoxon test. The probability level of $<0.05$ was taken as the limit of significant difference.

### Results

*Normal breast tissue*

The expression of c-*kit* proto-oncogene product was observed homogeneously in the cytoplasm and/or on cell membrane of alveolar and ductal mammary epithelia in all specimens (Figure 1a). The average IRS of normal breast tissue was $6.22 \pm 2.11$ (mean ± s.d.) (Table I).

*Benign breast tissue*

In benign breast tissue, the expression was found to be distributed heterogeneously in the cytoplasm or on the cell membrane of tumour cells (Figure 1b–d). The average IRS of c-*kit* proto-oncogene product expression tended to be reduced as $3.33 \pm 2.44$ in 58 benign breast diseases (Table I). Especially in intraductal papilloma of the breast, the IRS was significantly reduced to $1.34 \pm 1.70$, compared with that of fibrocystic change (IRS, $3.88 \pm 2.45$) or of fibroadenoma (IRS, $4.11 \pm 2.16$) (Table II).

*Breast cancer*

In breast cancer, the expression of c-*kit* proto-oncogene product was often deleted. Even in the tumour showing positive staining, the expression was observed in the cytoplasm and/or on the cell membrane of the limited number of cancer cells (Figure 2). The expression was found

**Table I** Expression of c-*kit* proto-oncogene product in normal epithelia and in breast tissue from patients with benign diseases and malignant tumours of the breast

| *Source of tissue* | *Number of tissues* | *IRS of* c-kit *expression (mean ± s.d.)* |
|---|---|---|
| Normal epithelium | 20 | $6.22 \pm 2.11$* |
| Benign breast tissue | 58 | $3.33 \pm 2.44$* |
| Breast cancer tissue | 57 | $0.43 \pm 1.27$* |

* $P = 0.0001$.

**Figure 1** Immunohistochemical expression of c-*kit* proto-oncogene product in human normal breast tissues and benign diseases. **(a)** Normal mammary epithelia. Bar = 25 μm. All of the epithelial cells expressed c-*kit* proto-oncogene product on plasma membrane or in the cytoplasm. **(b)** Fibrocystic change. The expression of c-*kit* proto-oncogene product was observed in the ductal epithelial hyperplasia in the lesion of fibrocystic change. Bar = 25 μm. **(c)** Intraductal papilloma. Bar = 50 μm. **(d)** Fibroadenoma. Bar = 50 μm. **b–d** represent the heterogeneous expression of c-*kit* proto-oncogene product in benign breast diseases.

to be restricted in cancer cells showing acinar differentiation (Figure 2c). The average IRS of breast cancer was only $0.43 \pm 1.27$ (Table I). The complete deletion was found in 40 of 57 (70.2%) breast cancer tissues. The IRS of c-*kit* proto-oncogene product expression in primary breast cancer was significantly lower compared to those of the normal breast tissues of the benign breast diseases ($P = 0.0001$) (Table I). To evaluate the changes of c-*kit* proto-oncogene product expression during malignant transformation in the human breast, the relationship between the IRS of normal duct, intraductal papilloma, non-invasive and invasive carcinoma was studied (Table III). The IRS of intraductal papilloma was significantly lower than that of normal duct. Although

**Table II** Expression of c-*kit* proto-oncogene product in benign diseases of the breast

| *Benign diseases* | *Number of tissues* | *IRS of* c-kit *expression (mean ± s.d.)* |
|---|---|---|
| Fibrocystic change | 23 | 3.88 ± 2.45* |
| Fibroadenoma | 20 | 4.11 ± 2.16** |
| Intraductal papilloma | 15 | 1.34 ± 1.70* |

* $P = 0.0013$; ** $P = 0.0003$.

**Figure 2** Immunohistochemical expression of c-*kit* proto-oncogene product in human breast cancer. (**a**) The staining of immunoreactive c-*kit* proto-oncogene protein was distributed focally in this breast cancer tissue. (**b**) Complete deletion of the expression of c-*kit* proto-oncogene product was observed in cancer cells. In contrast, diffuse staining was observed on the alveolar mammary epithelia in this specimen. (**c**) The expression of c-*kit* proto-oncogene product was detected on cancer cells showing acinar differentiation. Bar = 50 $\mu$m.

**Table III** Changes in the expression of c-*kit* proto-oncogene product in human breast diseases

| *Tissues* | *Number of tissues* | *IRS of* c-kit *expression (mean ± s.d.)* |
|---|---|---|
| Normal duct | 16 | 7.13 ± 1.82 |
| Intraductal papilloma | 15 | 1.34 ± 1.70 |
| Non-invasive carcinoma | 4 | 0.90 ± 1.55 |
| Invasive carcinoma | 50 | 0.41 ± .129 |

* $P = 0.0001$; † $P = 0.0277$.

**Table IV** Correlation between the expression of the c-*kit* proto-oncogene protein and the clinicopathological parameters of breast cancer

| *Parameters* | *Number of tissues* | *IRS of* c-kit *expression (mean ± s.d.)* | P-*value* |
|---|---|---|---|
| ER | | | |
| (–) | 31 | 0.51 ± 1.67 | |
| (+) | 26 | 0.36 ± 0.84 | NS |
| Menopausal status | | | |
| pre- | 26 | 0.44 ± 0.92 | |
| post- | 31 | 0.42 ± 1.52 | NS |
| Tumour size | | | |
| T1 | 20 | 0.55 ± 1.87 | |
| T2 | 25 | 0.40 ± 0.91 | |
| T3 | 8 | 0.43 ± 0.69 | NS |
| T4 | 4 | 0.00 ± 0.00 | |
| Histological | | | |
| Solid tubular | 29 | 0.46 ± 1.58 | |
| Papillotubular | 10 | 0.50 ± 1.01 | |
| Scirrhous | 11 | 0.22 ± 0.48 | NS |
| Non-invasive | 4 | 0.90 ± 1.55 | |
| Mucinous | 3 | 0.00 ± 0.00 | |
| Differentiation | | | |
| I | 19 | 0.41 ± 0.38 | |
| II | 23 | 0.69 ± 0.18 | NS |
| III | 15 | 0.38 ± 0.84 | |
| Lymph node | | | |
| metastasis | 27 | 0.56 ± 1.70 | |
| N0 | 15 | 0.75 ± 1.69 | |
| N1$\alpha$ | 8 | 0.09 ± 0.25 | NS |
| N1$\beta$ | 1 | 0.00 ± 0.00 | |
| N2 | 3 | 0.67 ± 1.16 | |
| N3 | | | |
| Distant metastasis | | | |
| M0 | 41 | 0.43 ± 1.40 | |
| M | 16 | 0.41 ± 0.91 | NS |
| TNM stage | | | |
| I | 29 | 0.53 ± 1.64 | |
| II | 11 | 0.21 ± 0.47 | |
| III | 1 | 0.00 ± 0.00 | NS |
| IV | 16 | 0.41 ± 0.91 | |

IRS, immunoreactive score; ER, oestrogen receptor; NS, not significant.

no significant difference was found between non-invasive and invasive carcinoma, the IRS of intraductal papilloma was higher than that of invasive carcinoma, and the intensity and the pattern of the staining were found to be similar to those seen in non-invasive carcinoma.

No significant relationship was found between the expression of the c-*kit* proto-oncogene product and the clinicopathological parameters of the breast cancer, such as grade of differentiation, tumour size, lymph node metastasis, distant metastasis, TNM stage, presence of ER, and menopausal status of the patient (Table IV). The patients were followed-up for three years in this study and no significant association was observed between the expression of this protein and the prognosis of patients with breast cancer.

### Discussion

The immunoreactive expression of c-*kit* proto-oncogene product was not observed in human normal lung or seminal vesicles tissue (Natali *et al.*, 1992*a*; Matsuda *et al.*, 1993), whereas it was found in 56% of SCLC (Matsuda *et al.*, 1993 and 80% of seminomas (Strohmeyer *et al.*, 1991). On the other hand, the expression was diffusely observed in human melanocytes, but, in primary melanomas, the deletion of the c-*kit* proto-oncogene product was observed in more invasive lesions (Natali *et al.*, 1992*c*). Previously a complete inverse pattern of the expression of c-*kit* proto-oncogene product has also been found in the normal tissues and the malignant tumours of the breast (Natali *et al.*, 1992*a*; Matsuda *et al.*, 1993).

In the present study the c-*kit* proto-oncogene product was uniformly expressed in normal breast tisses. In contrast, it was expressed heterogeneously in benign breast disease and the deletion was observed in breast cancer specimens with high incidence suggesting that the reduced c-*kit* proto-oncogene product expression is a general phenomenon in breast cancer. Previous studies have shown that direct cell–cell interaction between c-*kit* and its ligand, the membrane-bound form of stem cell factor (SCF), plays an important role in signal transduction (Flanagan, *et al.*, 1991; Reith *et al.*, 1991). Therefore, the high expression of the c-*kit* proto-oncogene product in normal breast tissue indicated that this protein may be related to the regulation of the proliferation and/or the differentiation of human normal mammary epithelia through the c-*kit* signalling pathway. Although it is possible to consider that the absence of staining in neoplastic cells is due to the presence of mutant c-*kit* product which is not reactive with the antibody used, the deletion of c-*kit* proto-oncogene product in breast cancer may indicate the changes of the signal transduction during malignancy in human mammary epithelia.

Recently, clonal analysis by means of polymerase chain reaction revealed that the intraductal papilloma was monoclonal in origin consisting of breast carcinoma, indicating that certain genetic changes had already occurred in the intraductal papilloma (Noguchi *et al.*, 1992, 1994). In the present study we found that the IRS of c-*kit* proto-oncogene product in intraductal papilloma was significantly reduced compared with that of other benign breast diseases. Since the loss of the expression of this protein could be considered to be related to the malignant transformation of human mammary epithelia (Natali *et al.*, 1992*b*), the observations in this study may indicate that the intraductal papilloma possesses higher malignant potential in benign breast diseases.

In this study no significant relationship was found between the expression of c-*kit* proto-oncogene product and the clinicopathological factors of breast cancer, although it is well known that the c-*kit* proto-oncogene product displays pleiotropic functions, such as the migration of the pigment stem cells (Keshet *et al.*, 1991) and the proliferation of the mast cells during normal development (Wershil *et al.*, 1992).

The reduction of c-*kit* proto-oncogene product expression observed in intraductal papilloma as well as breast cancer cells suggested that the deletion of c-*kit* proto-oncogene product would occur in the early phase of malignant transformation of human mammary epithelia. Further investigations will be required to clarify the active mechanism of c-*kit* proto-oncogene product in human breast.

### References

FLANAGAN JG, CHAN DC AND LEDER P. (1991). Transmembrane form of the kit ligand growth factor is determined by alternative splicing and is missing in the $Sl^d$ mutant. *Cell*, **64**, 1025–1035.

JAPANESE BREAST CANCER SOCIETY. (1992). *General Rules for Clinical and Pathological Recording of Breast Cancer*. 11th edn. Kanehara: Tokyo.

KESHET E, LYMAN SD, WILLIAMS DE, ANDERSON DM, JENKINS NA, COPELAND NG AND PARADA LF. (1991). Embryonic RNA expression patterns of the c-kit receptor and its cognate ligand suggest multiple functional roles in mouse development. *EMBO J.*, **10**, 2425–2435.

MATSUDA R, TAKAHASHI T, NAKAMURA S, SEKIDO Y, NISHIDA K, SETO M, SEITO T, SUGIURA T, ARIYOSHI Y, TALAHASHI T AND UEDA R. (1993). Expression of the c-kit protein in human solid tumors and in corresponding fetal and adult normal tissues. *Am. J. Pathol.*, **142**, 339–346.

NATALI PG, NICOTRA MR, SURES I, SANTORO E, BIGOTTI A AND ULLRICH A. (1992*a*). Expression of c-kit receptor in normal and transformed human nonlymphoid tissues. *Cancer Res.*, **52**, 6139–6143.

NATALI PG, NICOTRA MR, SURES I, MOTTOLESE M, BOTTI C AND ULLRICH A. (1992*b*). Breast cancer is associated with loss of the c-kit oncogene product. *Int. J. Cancer*, **52**, 713–717.

NATALI PG, NICOTRA MR, WINKLER AB, CAVALIERE R, BIGOTTI A AND ULLRICH A. (1992*c*). Progression of human cutaneous melanoma is associated with loss of expression of c-kit proto-oncogene receptor. *Int. J. Cancer*, **52**, 197–201.

NOGUCHI S, MOTOMURA K, INAJI H, IMAOKA S AND KOYAMA H. (1992). Clonal analysis of human breast cancer by means of polymerase chain reaction. *Cancer Res.*, **52**, 6594–6597.

NOGUCHI S, MOTOMURA K, INAJI H, IMAOKA S AND KOYAMA H. (1994). Clonal analysis of predominantly intraductal carcinoma and precancerous lesions of the breast by means of polymerase chain reaction. *Cancer Res.*, **54**, 1849–1853.

QIU F, RAY P, BROWN K, BARKER PE, JHANWAR S, RUDDLE FH AND BESMER P. (1988). Primary structure of c-kit: relationship with the CSF-1/PDGF receptor kinase family-oncogenic activation of v-kit involves deletion of extracellular domain and C terminus. *EMBO J.*, **7**, 1003–1011.

REITH AD, ELLIS C, LYMAN S, ANDERSON DM, WILLIAMS DE, BERNSTEIN A AND PAWSON T. (1991). Signal transduction by normal isoforms and W mutant variants of the kit receptor tyrosine kinase. *EMBO J.*, **10**, 2451–2459.

REMMELE W AND STEGNER HE. (1986). Immunohistochemischer Nachweis von Östrogenrezeptroren (ERICA) in Mammakarzinomgewebe: Vorschlag zur Einheitlichen Formulierung des Untersuchungsbefundes. *Dtsch. Ärztebl.*, **83**, 3362–3364.

STROHMEYER T, PETER S, HARTMANN M, MUNEMITSU S, ACKERMANN R, ULLRICH A AND SLAMON DJ. (1991). Expression of the hst-1 and c-kit protooncogenes in human testicular germ cell tumors. *Cancer Res.*, **51**, 1811–1816.

WERSHIL BK, TSAI M, GEISSLER W, ZSEBO KM AND GALLI SJ. (1992). The rat c-kit ligand, stem cell factor, induces c-kit receptor-dependent mouse mast cell activation in vivo. Evidence that signaling through the c-kit receptor can induce expression of cellular function. *J. Exp. Med.*, **175**, 245–255.